# A global approach to HIV-1 vaccine development

**DOI:** 10.1111/imr.12073

**Published:** 2013-06-16

**Authors:** Kathryn E Stephenson, Dan H Barouch

**Affiliations:** 1 Center for Virology and Vaccine Research, Beth Israel Deaconess Medical CenterBoston, MA, USA; 2 Ragon Institute of MGH, MIT, and HarvardBoston, MA, USA

**Keywords:** HIV, vaccines, viral diversity, immune responses

## Abstract

A global human immunodeficiency virus-1 (HIV-1) vaccine will have to elicit immune responses capable of providing protection against a tremendous diversity of HIV-1 variants. In this review, we first describe the current state of the HIV-1 vaccine field, outlining the immune responses that are desired in a global HIV-1 vaccine. In particular, we emphasize the likely importance of Env-specific neutralizing and non-neutralizing antibodies for protection against HIV-1 acquisition and the likely importance of effector Gag-specific T lymphocytes for virologic control. We then highlight four strategies for developing a global HIV-1 vaccine. The first approach is to design specific vaccines for each geographic region that include antigens tailor-made to match local circulating HIV-1 strains. The second approach is to design a vaccine that will elicit Env-specific antibodies capable of broadly neutralizing all HIV-1 subtypes. The third approach is to design a vaccine that will elicit cellular immune responses that are focused on highly conserved HIV-1 sequences. The fourth approach is to design a vaccine to elicit highly diverse HIV-1-specific responses. Finally, we emphasize the importance of conducting clinical efficacy trials as the only way to determine which strategies will provide optimal protection against HIV-1 in humans.

This article is part of a series of reviews covering HIV Immunology appearing in Volume 254 of *Immunological Reviews*.

## Introduction

A global human immunodeficiency virus-1 (HIV-1) vaccine will need to elicit durable, potent, and comprehensive immune responses to provide protection against highly diverse HIV-1 variants 1–2. There is more reason than ever to be hopeful about the development of an effective global HIV-1 vaccine. The RV144 trial conducted in Thailand demonstrated that an HIV-1 vaccine was capable of eliciting modest and transient protection against HIV-1 acquisition 3. A follow-up evaluation of the immune correlates of reduced HIV-1 risk in RV144 has revealed hopeful leads on how to improve vaccine efficacy, particularly in terms of the importance of Env-specific antibodies 4. Meanwhile, recent advances have allowed the discovery and characterization of broadly reactive neutralizing antibodies isolated from HIV-1-infected individuals 5–11. Recent studies of acute HIV-1 infection have also shown that the viral burden at the point of transmission may not be as formidable as first believed 12, involving single transmitter/founder viruses that may be easier to neutralize 13.

Given the vast diversity of HIV-1 worldwide, an effective global HIV-1 vaccine will need to provide protection against a diverse landscape of HIV-1 sequences in multiple demographic populations. Moreover, the development of a global HIV-1 vaccine will require large-scale clinical trials in human subjects. In this review, we first describe the current state of the HIV-1 vaccine field, outlining immune responses that may be desirable in a global HIV-1 vaccine. We then discuss select strategies for addressing the challenge of HIV-1 diversity.

## A global vaccine needs a global reach

While there are many challenges in HIV-1 vaccine development, a key hurdle is the tremendous genetic diversity of globally circulating strains of HIV-1 14–19. Because of the ability of HIV-1 to evade immune responses through mutational escape, there is constant viral evolution within populations and individual hosts. The genetic diversity of HIV-1 is attributable in part to the low fidelity of its reverse transcriptase, the large number of replication cycles of the virus, the influence of innate and adaptive immune responses, and the ability for HIV-1 to tolerate this diversity 14.

There are thirteen distinct HIV-1 subtypes and sub-subtypes that are linked geographically or epidemiologically, with within-subtype variation of envelope proteins of 15–20%, and between-subtype variation of up to 35% 14,15. Moreover, there are additional circulating recombinant forms (CRFs) generated from genetic mixing in persons dually infected with different subtypes. HIV-1 also diversifies extensively within each host. For example, Korber *et al*. 21 have demonstrated that the variability of HIV-1 within one host is comparable to the global variation of influenza A. This genetic diversity makes it difficult to design an HIV-1 vaccine that will be immunologically relevant in the face of such a variety of HIV-1 sequences.

### Env-specific antibodies to protect against HIV-1 acquisition

HIV-1 genetic diversity makes it particularly challenging to design a vaccine that can elicit broadly protective antibodies. There is an increasing consensus that antibodies specific to HIV-1 envelope (Env) will likely be required to block acquisition of HIV-1 22–23. Follow-up analyses of RV144 showed that antibodies to variable loops 1 and 2 (V1V2) regions of HIV-1 Env were associated with a reduced risk of HIV-1 acquisition 3,4. A recent genetic analysis provided further support for the importance of V2-specific antibodies by demonstrating that the RV144 vaccine had increased efficacy against viruses that matched the Env immunogen in the V2 location 25. Similarly, a recent study in non-human primates from our group demonstrated that SIV vaccines using adenovirus and poxvirus vectors afforded partial protection against neutralization-resistant SIVmac251 acquisition in rhesus monkeys, and that Env-specific antibodies were associated with decreased SIV infection risk 26. These vaccine-elicited antibodies included antibodies against V2 as well as other epitopes. Our group also demonstrated that Env was required to achieve significant protection against SIVmac251 challenge, and similar correlates have been reported by other laboratories against SIVsmE660 challenges 27–28.

Neutralizing Env-specific antibodies have also been shown to protect against HIV-1 and simian/human immunodeficiency virus (SHIV) acquisition in passive transfer experiments in non-human primates 29–39. For example, passive transfer of the neutralizing monoclonal antibodies 2F5, 2G12, and 4E10 was shown by Mascola, Hessell, and others 31–34 to afford partial protection against intravenous and mucosal SHIV challenge. In addition, Hessell, Burton, and colleagues 35,36 showed that high serum concentrations of the neutralizing monoclonal antibody b12 protected macaques from intravenous SHIV challenge and that low concentrations of b12 protected against low-dose intravaginal SHIV challenge. Monoclonal b12 has also been shown to protect against SHIV infection when given at high-doses intravaginally 37–38. More recently, the potent neutralizing antibody PGT121 was shown to protect against high-dose mucosal SHIV challenge in macaques at serum concentrations significantly lower than needed for protection in prior studies 39.

Non-neutralizing antibodies might also have the potential to afford partial protection against HIV-1 infection 40–41. Non-neutralizing antibodies include various effector functions mediated by the Fc region of the antibody, which triggers the innate immune system to destroy the virus or virus-infected cells. Follow-up analysis of RV144 showed that in participants with low serum immunoglobulin A (IgA) responses, high levels of antibody-dependent cellular cytotoxicity (ADCC) correlated with a reduced risk of HIV-1 infection 42. In addition, Liao and colleagues 43 recently demonstrated that V2-specific antibodies isolated from RV144 vaccines mediated ADCC against HIV-1-infected CD4^+^ T cells from RV144 subjects with breakthrough infections, and that this activity was dependent on V2 position 169 in breakthrough Envs. Theoretically, increased serum IgA responses might blunt the protective action of non-neutralizing antibodies because the IgA Fc region does not mediate the same effector functions, which might explain why serum IgA levels positively correlated with HIV-1 infection risk in RV144 vaccinees 4. It has also been shown that broadly neutralizing antibodies also rely on the Fc region for part of their protective activity 7.

The above studies suggest that Env-specific antibodies will likely be necessary to protect against HIV-1 acquisition. However, these studies do not directly address the challenge of HIV-1 diversity. For example, the immunogens used in RV144 matched the local Thai circulating strains of subtype B and the circulating recombinant form CRF01_AE 3. It is likely that the Env-specific antibodies elicited in RV144 would afford a lower degree of protection against other subtypes of HIV-1 found elsewhere in the world. However, it remains unclear how a vaccine can elicit antibodies that will recognize the substantial heterogeneity of Env sequences globally. Moreover, HIV-1 has other mechanisms to evade the humoral immune system, including low Env spike density on the virion surface, heavy glycosylation, conformational shielding of highly conserved Env epitopes, and mimicry of Env carbohydrates and proteins of host molecules 9–45.

### Cellular immune responses for virologic control

Given the challenges in eliciting broadly protective Env-specific antibodies, it is not likely that any vaccine would achieve 100% sterilizing immunity in all vaccinees; breakthrough HIV-1 infections will likely occur. Thus, it would be beneficial for an HIV-1 vaccine also to elicit immune responses capable of controlling viral replication 46. A wealth of the literature has shown that cellular immune responses can mediate control of viremia in HIV-1-infected humans and SIV-infected rhesus monkeys, including CD8^+^ T lymphocytes 47–57, NK cells 58, and CD4^+^ T lymphocytes 59–60. Moreover, vaccine trials in non-human primates have shown that sustained virologic control is achievable after heterologous SIV challenges. For example, our group has shown that adenovirus serotype 26 prime and modified vaccinia Ankara (MVA) boost expressing SIV antigens led to a 2.32 log reduction in mean set point viral load following stringent SIVmac251 challenge, and immune correlates of virologic control included the magnitude and breadth of Gag-specific cellular immune responses 26. Hansen *et al*. 56 also demonstrated that early profound and durable control of SIV replication were achieved in approximately half of rhesus monkeys immunized with a rhesus cytomegalovirus vector-based vaccine.

Whereas Env-specific antibodies appear necessary to block HIV-1 acquisition, Gag-specific cellular immune responses appear important for virologic control. For example, we have shown that Gag-specific CD8^+^ T cells correlated with both *in vivo* and *in vitro* virologic control following SIV challenge in vaccinated monkeys; no association was seen with Env- or Pol-specific CD8^+^ T cells 61. This result is consistent with studies demonstrating the association of Gag-specific cellular immune responses with virologic control in HIV-1-infected individuals 62–68 and SIV-infected rhesus monkeys 26–71. In addition to Gag, Vif and Nef may contribute to virologic control in certain settings, such as Mamu-B*08 monkeys 72.

Another critical aspect of cellular immune responses is the location and phenotype of cellular immune responses elicited by vaccination. For example, Fukazawa and colleagues 73 demonstrated that the degree of protection mediated by a live attenuated SIV vaccine strongly correlated with the magnitude and function of SIV-specific, effector T cells in lymph nodes. They also demonstrated that the maintenance of these protective T cells was associated with the persistent replication of vaccine virus in follicular helper T cells.

Despite these observations in non-human primates, virologic control has yet to be achieved in clinical trials of HIV-1 vaccines in human subjects. Neither VAX003/004, the Step study, nor RV144 showed significant impact on viral loads in vaccine recipients who became infected with HIV-1 3,74. However, there was evidence for immune selection pressure on breakthrough HIV-1 sequences in the Step study, suggesting that vaccine-elicited cellular immune responses can exert immunologically relevant biologic effects in humans 76.

## Strategies for a global HIV-1 vaccine

**Figure 1:**
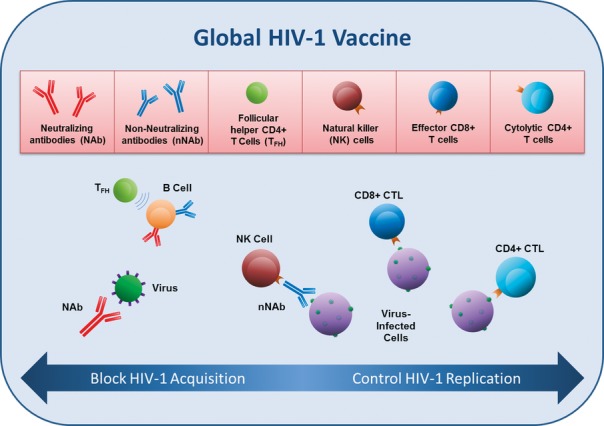
Immune responses targeted by a global HIV–1 vaccine.

The current state of HIV-1 vaccine research suggests that an effective global HIV-1 vaccine will need to elicit Env-specific antibodies to block HIV-1 acquisition and that these humoral immune responses will need to include either neutralizing or non-neutralizing antibodies (*Fig. *1). In addition, a global HIV-1 vaccine will need to elicit cellular immune responses to control viral replication for breakthrough HIV-1 infections. These cellular immune responses will most likely need to include Gag-specific CD8^+^ T cells.

It is unclear which HIV-1 antigens to include in a vaccine to address the challenge of global HIV-1 sequence diversity. Currently, there are four major strategies toward selecting antigens for a global HIV-1 vaccine (*Table 1*). The first approach is to design specific vaccines for each geographic region that include antigens tailor-made to match local circulating HIV-1 strains. The goal of these vaccines is to elicit HIV-1 subtype-specific immune responses that will have a higher likelihood of recognizing local strains. The second approach is to design a vaccine that will elicit Env-specific antibodies capable of broadly neutralizing all HIV-1 subtypes. The third approach is to design a vaccine that will elicit immune responses that are focused on highly conserved HIV-1 sequences. The rationale for this strategy is that these responses will recognize a multitude of different HIV-1 subtypes via a shared epitope target. The fourth strategy is to design a vaccine to elicit highly diverse HIV-1-specific responses. Here the rationale is that the greater the breadth and depth of HIV-1 epitopes recognized by vaccinees, the greater the chance that these immune responses will match the transmitting HIV-1 strain.

**Table 1:** Strategies to overcome the challenge of HIV-1 diversity

Vaccines to elicit regional HIV-1-specific immune responses
Subtype AE immunogens for Southeast Asia
Subtype C immunogens for South Africa
Subtype A immunogens for East Africa
Subtype B immunogens for United States and Europe
Vaccines to elicit broadly neutralizing antibodies
Env monomer immunogens
Env trimer immunogens
Scaffolded neutralizing antibody epitopes
Germline targeted immunogens
Vaccines to elicit highly conserved HIV-1-specific cellular immune responses
Conserved epitope immunogens
Conserved region immunogens
Sector immunogens
Vaccines to elicit highly diverse HIV-1-specific immune responses
Multi-clade immunogens
Mosaic immunogens

### Vaccines to elicit regional HIV-1-specific immune responses

One approach to overcoming the challenge of HIV-1 diversity is to design region-specific vaccines to elicit immune responses specific to local circulating HIV-1 strains. Such a region-specific vaccine strategy was adopted in RV144, which used immunogens that matched the local Thai circulating strains of subtype B and the circulating recombinant form CRF01_AE 3. As discussed above, it would not be likely that such a region-specific vaccine would be relevant in other regions with different subtypes. Such vaccines would therefore need to be tailored for specific regions of the world. For example, as a follow-up to RV144, ALVAC vectors and gp120 proteins are being produced with subtype C immunogens for future clinical trials in South Africa 12. In theory, similar vaccines could be developed for subtype B in North America and Europe, subtype A in east Africa, and so on. A limitation of this approach is that it would likely be very difficult to test and license multiple HIV-1 vaccines in different regions of the world. Moreover, many regions such as central Africa have multiple circulating subtypes, sub-subtypes, and CRFs in one area.

Even if it proves difficult to develop multiple region-specific vaccines, there is substantial interest in improving the RV144 vaccine for use in Thailand. As noted above, the ALVAC vector and gp120 protein used in RV144 provided 31% protection against HIV-1 acquisition. While this degree of protection was modest and transient, there is interest in improving the RV144 vaccine regimen for possible licensure in high-risk populations 12. It was noted that vaccine efficacy in RV144 appeared to decline from 60% at 1 year to 31% at 3.5 years, suggesting that increasing the frequency and number of booster doses might improve vaccine efficacy. One approach to building on RV144, therefore, is to use essentially the same vaccine regimen (ALVAC prime/gp120 boost) but expand the immunization schedule significantly; clinical trials in Thailand of such an approach are planned 12.

In addition to changing the RV144 vaccine schedule, there are also efforts to improve upon the ALVAC vector used in RV144. Like other poxviruses, ALVAC is well suited to be a vaccine vector because of its large genome (allowing for the integration of foreign DNA), thermostability, and the fact that genome replication occurs in the cytoplasm 77. It was advanced into Phase III trials in Thailand based on earlier clinical studies that showed that ALVAC vectors expressing subtype B Gag and CRF01_AE Env elicited antibody responses and cellular immune responses 12–78. Nevertheless, alternative poxvirus vectors, such as MVA and NYVAC, are candidates for replacing ALVAC in RV144-like vaccine formulations 12–84. Future studies using NYVAC vectors and gp120 protein boosts are planned for South Africa.

### Vaccines to elicit broadly neutralizing antibodies

A prominent but elusive aim of the field is to develop an HIV-1 vaccine that will elicit antibodies that can neutralize all circulating HIV-1 sequences 6–10. Neutralizing antibodies do not develop until late in natural infection and in only 10–30% of HIV-1-infected individuals 9–85. Until recently, the field was limited by relatively few broadly neutralizing monoclonal antibodies and a limited number of epitopic targets 6. However, recent developments in high throughput single-cell BCR-amplification assays have helped revolutionize the field, leading to the isolation and characterization of dozens of new broadly neutralizing monoclonal antibodies 11–90. To date, four highly conserved regions on HIV-1 Env are targeted by broadly neutralizing antibodies, including the CD4^+^ binding site (CD4bs), a quaternary site on the V1V2 loops, carbohydrates on the outer domain, and the membrane-proximal external region 9. For example, Walker and colleagues 11 described 17 new PGT antibodies that neutralize broadly across subtypes, some of which were 10-fold more potent than the broadly neutralizing antibodies PG9, PG16, and VRC01. In addition, Scheid and colleagues 87 identified a novel class of potent antibodies that mimic CD4 binding entitled ‘highly active agonistic CD4bs antibodies’, which include broadly neutralizing antibodies NIH45-46 and 3BNC117. Huang and colleagues have also described 10E8, a HIV-1 gp41 membrane-proximal external region-specific antibody that neutralized approximately 98 percent of tested viruses, which was non-self-reactive 90.

Despite the discovery of these remarkable broadly neutralizing monoclonal antibodies, it is still unclear how to elicit such antibodies by immunization. In fact, a major gap in the HIV-1 vaccine field is the absence of immunogens capable of eliciting neutralizing antibodies of substantial breadth. Many of the neutralizing monoclonal antibodies arise from extensive somatic mutation of heavy chains after years of chronic viral infection. The current effort to elicit broadly neutralizing antibodies via immunization uses the structure of previously identified neutralizing antibodies as a starting point for immunogen design. For example, several laboratories have used previously identified antibodies, such as the V1V2-directed antibodies PG9, PG16, and CH01-CH04, to screen for binding to gp120 envelope monomers which can then be developed into potential immunogen candidates 10. Other researchers have centered on designing Env immunogens to elicit antibodies that resemble the VRC01 antibody, which binds the highly conserved conformational CD4 binding site 10–44. Protein scaffolds have also been used to express neutralizing epitopes 91,92.

Env proteins might be trimers, monomers, or scaffolded neutralizing antibody epitopes, but all face the same challenge of achieving extensive somatic mutation seen in the broadly reactive neutralizing monoclonal antibodies isolated from chronically infected individuals. One approach to overcome this challenge is to design vaccines that target the germline precursors of the desired antibodies, and that aim to drive appropriate affinity maturation, so-called ‘B-cell-lineage vaccine design’ 45,86. Another novel approach is to use vector-mediated gene transfer to produce broadly neutralizing antibodies directly 95–96.

### Vaccines to elicit highly conserved HIV-1-specific cellular immune responses

A third approach to address the challenge of HIV-1 diversity is to design vaccines that will elicit cellular immune responses specific to highly conserved HIV-1 regions. The hypothesis underlying this strategy is that immune responses specific for conserved HIV-1 regions will recognize a multitude of different HIV-1 subtypes as the diverse strains all share a common highly conserved epitope target and that these immune responses will impose a high fitness cost on any HIV-1 escape viral mutants 18–99. An initial emphasis was on selecting natural sequence antigens that may be most conserved among circulating HIV-1 strains. The Step study adopted this approach, using an adenovirus serotype 5 vector to express subtype B Gag, Pol, and Nef sequences that were selected to be phylogenetically close to consensus B sequences 100–101. There is evidence that this vaccine exerted immune selection pressure on breakthrough HIV-1 sequences, but cellular immune breadth was narrow and insufficient to mediate virologic control 76.

Several other HIV-1 vaccine immunogens have been designed with the goal of inducing responses against conserved epitopes, with varying success in preclinical and clinical studies. For example, the HIVA immunogen, first described in 2000, was derived from the p24 and p17 segments of HIV-1 clade A Gag fused to a string of 25 partially overlapping cytotoxic T-lymphocyte (CTL) epitopes 102. HIVA was shown to induce multiple HIV-1-specific CTL epitopes when expressed by DNA and MVA vectors in rhesus monkeys 103 and in a small phase 1 clinical trial in humans 104. However, when the HIVA immunogen was tried in larger clinical trials, the immunogen induced minimal HIV-1-specific T lymphocyte responses 105. Similarly, the EP HIV-1090 immunogen, first described in 2003 as part of a DNA vaccine, was derived from 21 CTL epitopes of HIV-1 that bound multiple HLA types and represented conserved sequences from multiple HIV-1 subtypes 106. When tested in humans, EP HIV-1090 and a later version, EP-1233, were both poorly immunogenic 80–107. These studies suggested that polyepitope immunogens were not optimally processed or presented by human immune systems and were not good candidates for inducing conserved-specific T lymphocyte responses.

Another strategy is to include longer fragments of conserved HIV-1 regions, as is done in the immunogen HIV_consv_. When expressed by DNA and viral vector vaccines, HIV_consv_ has been shown to be immunogenic in preclinical studies 108–109. However, we have shown in non-human primates that at least in certain situations full-length HIV-1 immunogens elicit increased magnitude and breadth of cellular immune responses compared with conserved-region-only HIV-1 immunogens 110. Phase 1 clinical trials of the safety and immunogenicity of HIV_consv_ are ongoing (NCT01151319 and NCT01024842).

A variation of this approach is to design immunogens based strictly on conserved HIV-1 segments with mutable regions excluded completely 111. In contrast to the sequences in HIVA and EP-1033, the conserved sequences included in these immunogens do not necessarily have to correspond to any known T-cell epitope. Similarly, Dahirel and colleagues proposed designing immunogens based on HIV-1 sequence sectors that exhibit higher order conservation as measured by random matrix theory 62. These are sectors in which multiple mutations are very rare, suggesting they are regions of immunologic vulnerability.

### Vaccines to elicit highly diverse HIV-1-specific immune responses

The above vaccine strategies share a common goal to elicit immune responses specific to highly conserved HIV-1 regions, with the hypothesis that these responses will recognize conserved sequences shared by a wide variety of HIV-1 strains. A contrasting strategy is to design vaccines that elicit diverse immune responses specific for a broad array of HIV-1-specific sequences. The hypothesis underlying this strategy is that the greater and more diverse the immune responses, the greater the likelihood that there will be a match to the transmitting HIV-1 strain. Diverse immune responses include T-cell and B-cell specificities that recognize multiple HIV-1 regions (breadth) and also multiple variants of HIV-1 epitopes for each epitopic locus (depth).

Currently, there are two primary approaches for eliciting broad HIV-1-immune responses via vaccination. The first is to design multivalent immunogens that represent multiple different HIV-1 clades 112. For example, the U.S. Military HIV Research Program has developed HIV-1 immunogens based on the predominant HIV-1 subtypes in Kenya, Tanzania, Uganda, and Thailand 113, and a recent Phase I clinical study showed that this vaccine was well tolerated and elicited durable cell-mediated and humoral immune responses 82. Similarly, a phase II clinical trial sponsored by the HIV Vaccines Trial Network (HVTN 505, NCT00865566) tested the DNA prime/Ad5 boost vaccine expressing Env proteins from subtypes A, B, and C developed by the NIH Vaccine Research Center.

The second approach to eliciting broad immune responses is the design of so-called ‘mosaic’ immunogens 114. These immunogens are engineered by *in silico* analysis of global HIV-1 sequences to provide maximal coverage of viral sequence diversity 115. Several laboratories have shown that mosaic HIV-1 immunogens elicited a greater breadth and depth of HIV-1 cellular immune responses than consensus or natural HIV-1 immunogens in non-human primates, as well as comparable or improved Env-specific binding and neutralizing antibody responses 116–117. Moreover, full-length mosaic HIV-1 immunogens elicited greater immune responses than conserved-region-only HIV-1 immunogens 110. Based on these data, mosaic immunogens are progressing into clinical development, in the context of Ad26 and MVA vectors expressing mosaic HIV-1 Gag, Pol, and Env immunogens, as well as in DNA and NYVAC vectors expressing mosaic HIV-1 Env immunogens.

The vaccines described above are intended to be global vaccine concepts. Vaccine delivery vehicles will also need to be globally relevant. One strategy to avoid the problem of anti-vector immunity is to use plasmids containing HIV-1 DNA sequences, such as VRC-HIVDNA016, a 6-plasmid multiclade HIV-1 DNA vaccine used in HVTN 505 118–119. DNA vaccines can further be improved by electroporation 118. Another strategy to minimize anti-vector immunity is to use lower seroprevalence or non-human viruses as vectors 120–125. For example, the lower seroprevalence and low titer adenoviruses such as adenovirus serotype 26 (Ad26) and Ad35 are now being studied as HIV-1 vaccine vectors 120,121. Preclinical studies have shown that these adenoviruses have significant biologic differences from Ad5, the vector used in the Step study that suggested a possible increased risk of HIV-1 acquisition in the subset of vaccinees with baseline anti-vector antibodies 101–133. Ad26 and Ad35 vectors are therefore planned for further clinical development 134–135.

## Conclusions

The tremendous global diversity of HIV-1 poses one of the greatest challenges for the development of an effective global HIV-1 vaccine. Recent research has underscored the importance of Env-specific antibodies for blocking HIV-1 acquisition and CD8^+^ T lymphocytes for mediating virologic control, yet the optimal strategy for confronting HIV-1 sequence diversity remains unknown. Here we have outlined four key strategies for developing a global HIV-1 vaccine, that is, to design vaccines that elicit (i) region-specific immune responses; (ii) broadly neutralizing antibodies; (iii) highly conserved cellular immune responses; or (iv) highly diverse immune responses. The only way to define which of these strategies will provide optimal protection against HIV-1 in humans will be to test a subset of the most promising vaccine strategies in clinical efficacy trials. By confronting the challenge of HIV-1 sequence diversity, the field can move closer to an effective global HIV-1 vaccine.
